# Improved Isolation Optimizes Downstream Application of Extracellular Vesicles Derived from *Mycobacterium tuberculosis*

**DOI:** 10.3390/microorganisms12112129

**Published:** 2024-10-24

**Authors:** Wenjing Wang, Yue Hou, Jingfang Zhang, Zhaogang Sun, Hong Sun

**Affiliations:** 1 Beijing Chest Hospital affiliated to Capital Medical University, Beijing 100000, China; wwj28107@163.com (W.W.); hou9702@126.com (Y.H.); zhangjingfang@bjxkyy.cn (J.Z.); 2 Beijing Tuberculosis and Thoracic Tumor Research Institute, Beijing 100000, China

**Keywords:** mycobacteria, exosome detection via an ultrafast-isolation system, functional study, blood–brain barrier

## Abstract

*Mycobacterium tuberculosis* (*Mtb*), the causative agent of tuberculosis, secretes extracellular vesicles (EVs), which may play an important role in mediating interactions between bacteria and host cells. *Mtb* EVs can be isolated by means of various techniques, which differ in terms of their effectiveness. In the present study, we found that an exosome isolation kit (EI) yielded higher numbers of EVs than either differential centrifugation (DC) or exosome detection via an ultrafast-isolation system (EXODUS). We also found that the EXODUS method revealed a greater abundance of H37Rv components within EVs, compared with the DC and EI methods. Analysis of the downstream application of H37Rv EVs revealed their internalization by RAW264.7 macrophages, peaking at 6 h, with subsequent activation of the TLR2 signaling pathway leading to the expression of inflammatory cytokines including IL-6 and TNF-α. It was also found that H37Rv EVs could cross the blood–brain barrier (BBB) and enter the brain, peaking at 12 h post-injection, eliciting an inflammatory response in the cerebral parenchyma, cerebellum, and hippocampus that persisted for up to 6 days. These findings offer novel insights into the pathogenesis of *Mtb*-induced diseases and may guide the development of therapeutic strategies.

## 1. Introduction

Tuberculosis (TB), which is caused by *Mycobacterium tuberculosis* (*Mtb*), occurs in every country and remains one of the leading infectious causes of death worldwide [1]. According to a World Health Organization (WHO) report, in 2022, there were 7.5 million reported cases of TB [2]. The pathogenesis of *Mtb* has developed intricate and diverse mechanisms to manipulate host cellular physiology and evade the immune system [3]. Many of these interactions with hosts are largely mediated by mycobacterial cell envelope components, which are now understood to be exported within extracellular vesicles (EVs) [4]. Compared to non-pathogenic bacteria, pathogenic bacteria produce a greater number of vesicles [5]. EVs released by pathogenic *Mtb* contain immunomodulatory molecules and factors associated with mycobacterial virulence to enhanced bacterial replication within the infected tissues [6]. Moreover, EVs from *Mtb* can also elicit an immune response equivalent to BCG vaccination, potentially bolstering the protective effects of BCG [7,8]. Preliminary research on *Mtb* EVs has examined their biogenesis, immunological effects, and use in vaccines and diagnostics. Considering the critical role of *Mtb* EVs in facilitating medical diagnosis, prognosis, and understanding the pathogenic mechanisms of *Mtb*, it is imperative to establish a refined, reliable, and efficient method for isolating EVs from culture media.

Many isolation methods for EVs have been established, including ultracentrifugation, ultrafiltration, commercial kits, novel immunoaffinity capture methods, and microfluidic technology [9]. The choice of method for the isolation of EVs affects the yield, particle size, and protein quality of the EVs obtained, impacting downstream applications, especially functional studies [10]. However, to date, only a few methods have been reported for the isolation of *Mtb* EVs, including ultracentrifugation, density gradient centrifugation [1], ultrafiltration [11], and size-exclusion chromatography [12]. Moreover, there has yet been no comparative study of methods for isolating *Mtb* EVs, and the impact of these methods on downstream application studies remains unknown. Therefore, a comparative study of isolation methods for *Mtb* EVs is necessary to identify the most effective techniques for isolating high-quality *Mtb* EVs. The H37Rv strain, known for its potent virulence, is universally recognized as the global standard for *Mtb* research and is extensively utilized across laboratory settings [13]. For this study, we used ultracentrifugation (DC), a commercial isolation kit (EI), and microfluidic chips, specifically the EXODUS system, to isolate H37Rv EVs. We compared the differences among the isolated EVs and conducted a preliminary investigation on their downstream applications. Our findings have significant implications for advancing *Mtb* EVs-related research and elucidating the pathogenesis and treatment of TB.

## 2. Materials and Methods

### 2.1. Cultivating H37Rv and Extracting EVs Using Various Isolation Methods

The *Mtb* strain of H37Rv (ATCC27294) from the Beijing Bio-Bank of Clinical Resources on Tuberculosis was thawed and cultured in Sauton broth for 21 days at 37 °C. The culture was centrifuged, then filtered through a 0.22 μm filter; the filtrate was then collected in a sterile container, with all work carried out under biosafety-approved protocols in a Biosafety Level 2 (BSL-2) laboratory [14,15].

**Figure 1:**
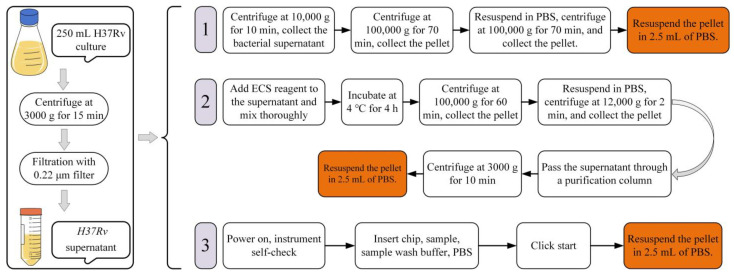
An overview of H37Rv EV isolation. 1: differential centrifugation; 2: exosome isolation kit (Solarbio, Beijing, China); 3: exosome detection via an ultrafast-isolation system.

H37Rv EVs were isolated using three methods: (1) differential centrifugation (10,000× *g* & 100,000× *g*) with equipment from Thermo Fisher Scientific (Waltham, Massachusetts, USA) [16], (2) an Exosome Isolation kit (EX0011, Solarbio, Beijing, China) (http://www.solarbio.com) (accessed on 21 February 2023), and (3) an ultrafast-isolation system (EXODUS H600, HUIXIN LIFETECH, Shenzhen, China) with L model chip [17]. After each method, EVs were stored at −80 °C. The three isolation methods are depicted schematically in Figure 1.

### 2.2. Transmission Electron Microscopy

The sample suspension was applied to a copper grid with a supporting film (AZH200, Zhongjingkeyi Technology, Beijing, China). Excess 2% phosphotungstic acid (12501-23-4, SPI-chem, Wilmington, DE, USA) was removed with absorbent paper, and staining solution applied for 3–5 min. Afterwards, the stain was blotted off, and the sample was dried completely, prior to observation via transmission electron microscopy (TEM) (HT7700, HITACHI, Tokyo, Japan).

### 2.3. Nanoparticle Tracking Analysis

After calibrating the nanoparticle tracking analysis (NTA) (ZetaView, Particle Metrix, Inning am Ammersee, Germany), the EV sample was diluted with 1 × PBS to 50–400 particles. The instrument parameters were then adjusted, and the analysis was initiated.

### 2.4. Protein Analysis

The total protein concentration was determined using the BCA Protein Assay Kit (P0010, Beyotime, Shanghai, China). The procedure followed the manufacturer’s instructions. Protein electrophoresis was run on a 12.5% SDS-PAGE gel, stained with Coomassie Brilliant Blue R250 (PS111, Epizyme Biomedical Technology, Shanghai, China); this was destained until bands were clear. The gel was then photographed. The Western blot protocol was adapted from Gang Chen [18]. The membrane was probed with primary antibodies PPD (20-MR50-1 mg, Universal Biotech, Shanghai, China), MPT64 (CSB-PA14947A0Rb, Cusabio, Wuhan, China), and TLR2 (EPR20302-119, Abcam, England) (all diluted 1:2000) overnight at 4 °C. After washing with PBST, the membrane was incubated with HRP-conjugated anti-rabbit IgG (GB23303, Servicebio, Wuhan, China) (1:3000) for 1 h at room temperature, and the immunoblot was visualized by means of chemiluminescence scanning.

Mass spectrometry (MS) analysis of H37Rv EVs was performed according to previously published procedures with some modifications [19] using an Orbitrap Exploris 480 mass spectrometer coupled to an EASY-nLC 1200 liquid chromatography system (Thermo Fisher Scientific, Waltham, MA, USA) in data-independent acquisition (DIA) mode. The DIA raw data files were analyzed using DIA-NN software (version 1.8).

### 2.5. Test of EV Uptake AND Viability in RAW264.7

H37Rv EVs (100 μg) were labelled with DiI (5 μmol), a lipophilic carbocyanine dye possessing a lengthy lipophilic alkyl chain for stable staining of the entire membrane [20], for 10 min at 37 °C in the dark. The labeled EVs were centrifuged at 120,000× *g* for 70 min to remove excess dye, and then washed [21]. RAW264.7 cells (CL-0190, Procell, Wuhan, China) were exposed to 50 μg of labelled H37Rv EVs for 1–8 h at 37 °C, and subsequently, the cell nuclei were stained with Hoechst 33342. Using an FV1000 confocal laser scanning microscope (Olympus, Hamburg, Germany), immunofluorescence images were captured at an image magnification of 400× with Exc/Em wavelengths of 549/565 nm and 346/460 nm, respectively. PBS DiI dye treatments were used as controls.

RAW264.7 cells were seeded at 1 × 10^4^ cells per well in 96-well plates overnight, and then treated with 5 μg H37Rv EVs for 24 h. A Cell Counting Kit-8 (CCK-8) (HY-K0301, MedChemExpress, Monmouth Junction, NJ, USA) was added for 1 h before the absorbance was measured at 450 nm using a Multiskan GO spectrophotometer (Thermo Fisher Scientific, Waltham, MA, USA).

### 2.6. Quantitative Real-Time PCR

RNA was extracted post-cell lysis with a Vazyme RNA Fast Extraction Kit (RC112, Nanjing, China) and assessed for concentration and quality using a Nanodrop 2000 (Thermo Fisher Scientific, Waltham, MA, USA). Reverse transcription was performed with a Fast Quant RT Kit with gDNase (KR106, Tiangen, Beijing, China), followed by amplification with PowerUp SYBR Green Master Mix (Applied Biosystems, Waltham, MA, USA) on a QuantStudio™ 12k Flex Real-Time PCR System (Applied Biosystems, San Diego, CA, USA). The PCR conditions comprised UDG activation at 50 °C for 2 min, pre-denaturation at 95 °C for 2 min, and 40 cycles of amplification at 95 °C for 30 s, 58 °C for 15 s, and 72 °C for 1 min.

Relative mRNA expression of *TLR2*, Interleukin-6 (*IL-6*), and tumor necrosis factor alpha (*TNF-α*) was calculated by the 2^−∆∆Ct^ method, with Ct > 35 considered as unexpressed. Primers were synthesized by Sangon Biotech (Shanghai, China), and their sequences are listed in Table S1.

### 2.7. ELISA

The cell culture supernatant was centrifuged at 1000× *g* for 20 min. Next, IL-6 and TNF-α (SEKM-0007 and SEKM-0034, Solarbio, Beijing, China) were measured using ELISA kits according to the manufacturer’s protocol, with absorbance readings taken at 450 nm using a Multiskan SkyHigh spectrophotometer (Thermo Fisher, MA, Waltham, USA).

### 2.8. Animal Experiments

Adult male ICR mice (6–8 weeks old, 18–20 g, Beijing Vital River Laboratory Animal Technology, Beijing, China) were housed in a specific pathogen-free environment. The in vivo manipulations were approved by the Institutional Animal Care and Use Committee of Beijing Tuberculosis and Thoracic Tumor Research Institute (Number: XK2023-084, XK2023-055).

H37Rv EVs (0.2 mg/pce) were injected into the tail veins of C57BL/6 mice, which were then euthanized by means of vertebral dislocation after 3, 6, and 10 days. Brain tissues were isolated and stained with hematoxylin and eosin (H&E) to observe any pathological changes.

H37Rv EVs labeled with DiI (0.2 mg/pce) were injected into nude mice intravenously. Near-infrared fluorescence images of the mice’s bodies and ex vivo organs were acquired at 1, 4, 8, 12, and 24 h. After homogenization, the fluorescence values of both liver and brain homogenates were measured using a Multifunctional Enzyme Labeler (Infinite 200, TECAN, Switzerland), with excitation set at λexc = 549 nm and emission detected at λem = 580 nm.

### 2.9. Statistical Analysis

All experiments were conducted with three independent biological replicates and reported as mean ± SD. Statistical analysis was performed using a two-tailed unpaired *t*-test in Prism (GraphPad, La Jolla, CA, USA). Significant differences (*p* < 0.05) are denoted in the respective graphs.

## 3. Results

### 3.1. Comparative Analysis of H37Rv EVs Isolated via Three Methods

#### 3.1.1. Electron Microscopy Analysis

**Figure 2:**
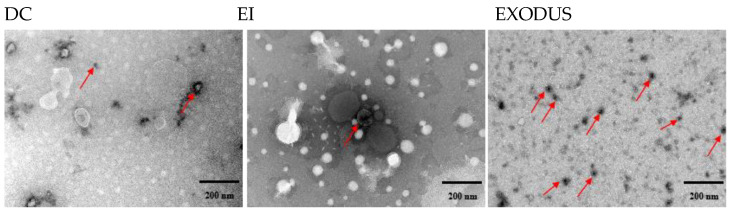
Morphological images of H37Rv EVs isolated by various methods under TEM. Scale bar: 200 nm. Arrows show typical EVs.

Three isolation methods were used to isolate H37Rv EVs and compare their efficiency: DC, EI following the manufacturer’s protocol, and an instrument-based protocol as per the manual (Figure 1). For each method, an identical volume of H37Rv culture supernatant (250 mL) was processed for EV isolation, with the final EVs resuspended in an equal volume of 1 × PBS (2.5 mL). Transmission electron microscopy (TEM) analysis of H37Rv EVs isolated by DC, EI, and EXODUS showed spherical particles with diameters of <200 nm. Additionally, TEM indicated that EVs isolated with EXODUS contained fewer impurities than those isolated with the EI kit (Figure 2).

#### 3.1.2. Nanoparticle Tracking Analysis

NTA precisely measures the size distribution of polydisperse samples by tracking individual particles, offering high resolution [22,23]. Table 1 presents the detailed characteristics of particle concentrations and sizes for three distinct categories: DC, EI, and EXODUS. Each category is characterized by an original concentration of particles per milliliter, which is then diluted by a specified factor to reach a final concentration. The table also includes the measured diameter of the particles in nanometers, as well as the percentiles (X_10_, X_50_, and X_90_) that describe the distribution of particle sizes within each category.

NTA of H37Rv EVs suspensions isolated by different methods revealed a consistent size range of less than 200 nm across all methods. The mean diameters of H37Rv EVs isolated via the EI, DC, and EXODUS methods were 149.4 nm, 135.0 nm, and 112.4 nm, respectively (Table 1, Figure S1). The EI method yielded the highest number of H37Rv EVs particles; indeed, this number was an order of magnitude higher than the numbers of EVs particles isolated using the EXODUS and DC methods (Table 1).

#### 3.1.3. Protein Analysis

**Figure 3:**
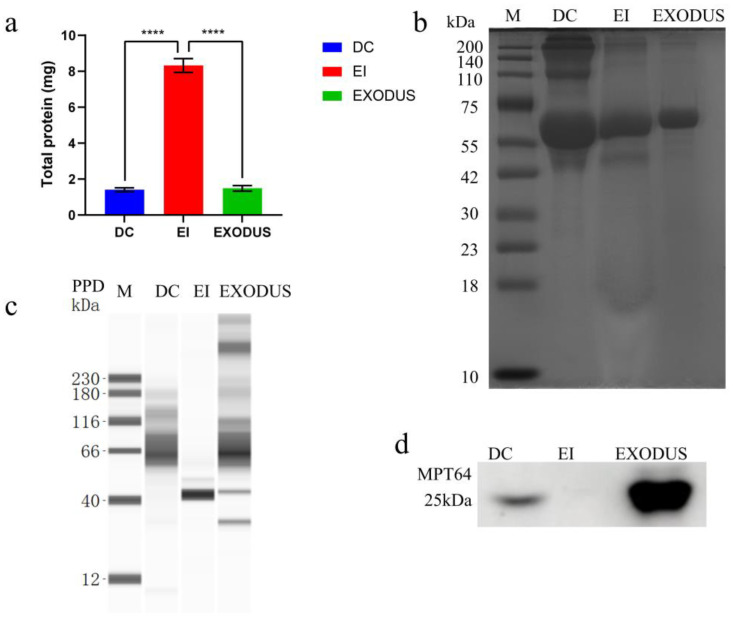
Protein content and composition of H37Rv EVs isolated by three methods. (**a**) Protein content of H37Rv EVs (n = 3). (**b**) Coomassie brilliant blue-stained gel image. (**c**) PPD protein immunoblot image. (**d**) MPT64 protein immunoblot image. M: protein marker; DC: differential centrifugation; EI: exosome isolation kit; EXODUS: exosome detection via an ultrafast-isolation system. **** *p* < 0.00001.

The total EVs yield was quantified by protein concentration with the EI method yielding the highest (8.4583 mg), followed by EXODUS (1.4833 mg), and DC (1.4083 mg) (Figure 3a). Coomassie brilliant blue staining revealed both shared and unique proteins in H37Rv EVs isolated by the three methods (Figure 3b; original gels in Figure S2a).

Total protein analysis and Coomassie brilliant blue staining could not distinguish between H37Rv proteins and other miscellaneous protein fractions. In light of this, we carried out protein immunoblotting of the three types of EVs for purified protein derivative (PPD), a complex protein mixture containing various antigenic components of *Mtb* [24,25], and the secreted protein MPT64, a specific antigen that plays a crucial role in *Mtb* infection [26,27]. The immunoblotting of PPD revealed a greater variety of *Mtb* proteins in EVs isolated using EXODUS, compared to the other two methods (Figure 3c). Additionally, H37Rv EVs isolated using the EI kit exhibited negative staining of MPT64 antibodies, indicating (Figure 3d) that, using the EI method, there was a considerable loss of certain *Mtb* components during the isolation process. The original blot scans are presented in Figure S2b–d.

Because the H37Rv EVs isolated using EXODUS contained more *Mtb* components, they were selected for MS analysis. This analysis identified 119 proteins, including lipoproteins, antigenic proteins, and those associated with secretion, transport systems, and stress responses (Table S2). Notably, lipoproteins accounted for 15.13% of the total identified proteins, a proportion significantly higher than previously reported enrichment of up to 10% among the 48 proteins identified in Mtb EVs [6,28]. Additionally, our MS analysis of H37Rv EVs revealed that many of these lipoproteins, such as LprA, LpqH, and LprG, are ligands for TLR2, which facilitates the delivery of immunologically active *Mtb* virulence factors [6,29].

### 3.2. Analysis of H37Rv EVs for Downstream Application

#### 3.2.1. Uptake of H37Rv EVs by RAW264.7 Cells

**Figure 4:**
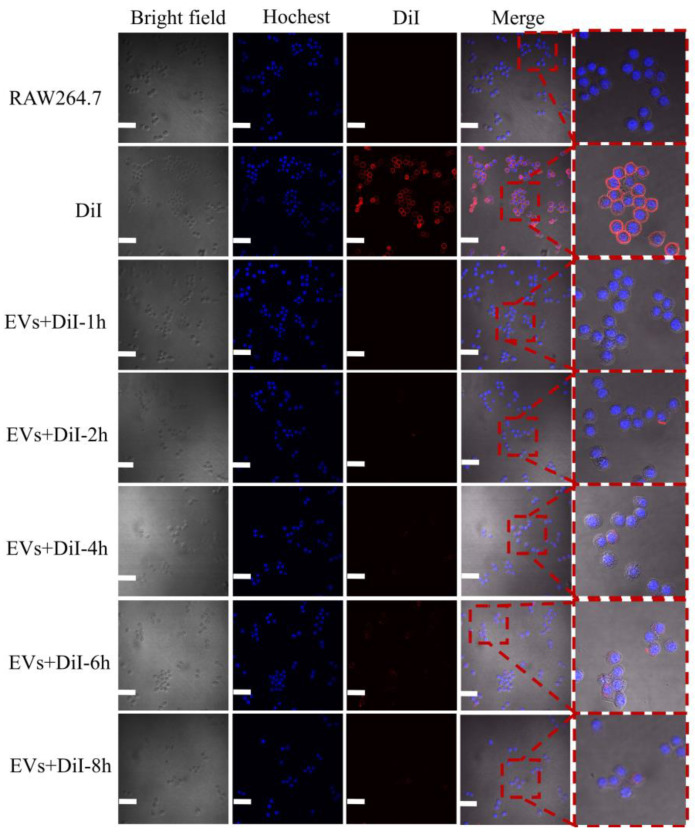
Uptake of H37Rv EVs by RAW264.7 cells in culture. Confocal microscopy showing uptake of H37Rv EVs after incubation for 1–8 h. H37Rv EVs appear in red, nuclei in blue. Scale bar: 50 μm.

EVs derived from bacteria are crucial in pathogenesis in activating intracellular signaling via ligand–receptor interactions and/or being internalized by the target cell [30,31,32]. In this study, we investigated the internalization of H37Rv EVs by RAW264.7 mouse macrophage cells, utilizing fluorescent labeling with the red membrane dye DiI. Our observations revealed that the uptake of these labeled EVs by RAW264.7 cells was not only variable but also time-dependent, exhibiting a gradual increase that peaked at the 6 h mark (Figure 4). Surprisingly, the intracellular concentration of H37Rv EVs decreased at 8 h, indicating that the initial rate of EVs internalization by RAW264.7 cells surpassed their expulsion. As the EVs continued to accumulate, exocytosis emerged as the dominant process, resulting in the extrusion of vesicles.

#### 3.2.2. Analysis of Immunological Effects of H37Rv EVs on RAW264.7 Cells

**Figure 5:**
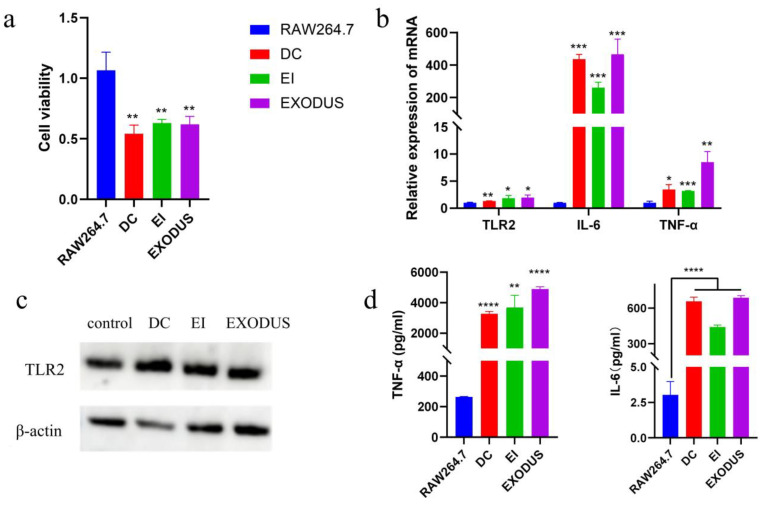
Analysis of immunological effects in RAW264.7 cells stimulated by H37Rv EVs. (**a**) Cell viability (n = 3). (**b**) Relative expression of *TLR2*, *IL-6* and *TNF-α* mRNA (n = 3). (**c**) Protein immunoblot image of TLR2. (**d**) Levels of IL-6 and TNF-α in the cell culture supernatant (n = 3). Control—RAW264.7; DC: differential centrifugation; EI: exosome isolation kit; EXODUS: exosome detection via an ultrafast-isolation system. *p* > 0.05, * *p* < 0.05, ** *p* < 0.001, *** *p* < 0.0001, **** *p* < 0.00001.

Because H37Rv EVs contain agonists of TLR2, the immune-inflammatory response of the TLR2 signal pathway in RAW264.7 cells was investigated after the internalization of H37Rv EVs. The CCK-8 assay was utilized to assess the impact of H37Rv EVs on the viability of RAW264.7 cells 24 h after post-induction. As depicted in Figure 5a, it is evident that, irrespective of the three isolation methods employed, the viability of the cells was significantly diminished after 24 h of incubation with 5 μg of H37Rv EVs protein per well. Furthermore, the H37Rv EVs isolated through various methods activated the expression of TLR2 (Figure 5b,c) and cellular inflammatory factors such as IL-6 and TNF-α (Figure 5b,d). Notably, EXODUS exhibited a higher capacity to stimulate the expression of inflammatory factors in RAW264.7. Specifically, qPCR analysis revealed that EXODUS induced a 1.96-fold increase in *TLR2*, a 465.75-fold increase in *IL-6*, and an 8.3-fold increase in *TNF-α* compared to controls (Figure 5b). ELISA results further emphasized this, showing that EXODUS elevated IL-6 expression by 227.46-fold and TNF-α expression by 18.58-fold (Figure 5d). Although the DC and EI methods also resulted in increases, their effects were less pronounced.

#### 3.2.3. Effects of H37Rv EVs on the Brain and Their Ability to Cross the Blood–Brain Barrier

**Figure 6:**
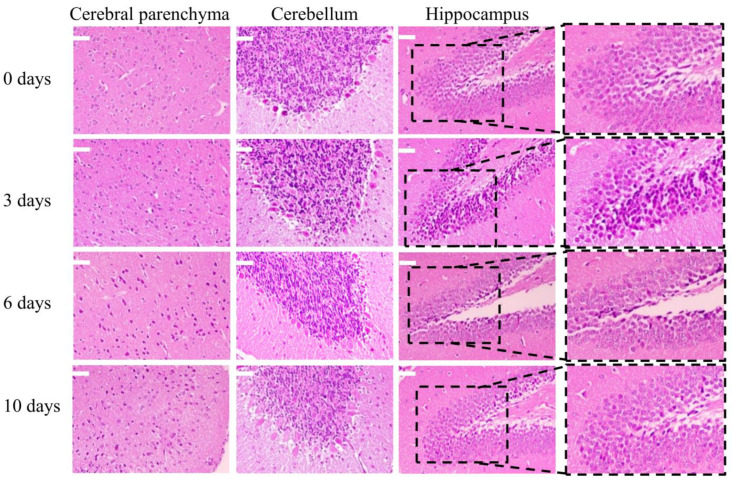
H&E staining of mouse brain at 0, 3, 6, and 10 days after injection of H37Rv EVs. There were three mice in each group. Scale bar: 50 µm.

The H37Rv EVs isolated from EXODUS, which exhibited the most pronounced inflammatory response in RAW264.7 cells, were chosen to investigate their impact on the mouse brain. After intravenously administering 200 μg of H37Rv EVs to C57BL/6 mice, we monitored pathological changes in brain tissue using H&E staining at 0, 3, 6, and 10 days after injection. On day 0, neurons with clear, well-structured nuclei were observed. By day 3, degeneration and intense staining were evident, particularly in the cerebellum and hippocampus. On day 6, the staining intensified, and cell spaces widened, although degeneration was less pronounced. By day 10, the staining persisted, but degeneration and structural degradation were further diminished compared to day 6. These findings suggest that H37Rv EVs may initiate brain inflammation (Figure 6).

**Figure 7:**
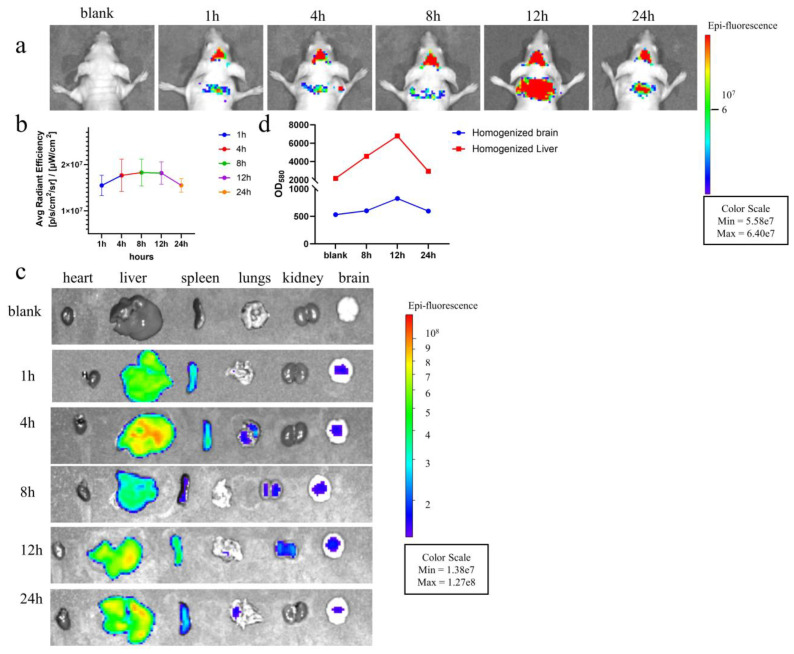
Distribution of H37Rv EVs in vivo in mice. (**a**) In vivo fluorescence images of nude mice from 1 to 24 h after intravenous administration of DiI-labeled H37Rv EVs. (**b**) Fluorescence value statistics (corresponding to (**a**)) of isolated brains from 1 h to 24 h after intravenous administration of DiI-labeled H37Rv EVs. (**c**) Ex vivo fluorescence image of isolated organs (including the brain) from 1 h to 24 h after intravenous administration of DiI-labeled H37Rv EVs. (**d**) Fluorescence value statistics images of homogenized brain and liver tissues (excitation = 549 nm, emission = 580 nm).

EVs possess robust penetration capabilities, and the brain inflammatory response induced by the H37Rv EVs may be attributed to their ability to reach the mouse brain. The BBB is a specialized protective barrier composed of brain microvascular endothelial cells, astrocytes, and pericytes. This ensures the integrity of the neural microenvironment and shields the brain from bloodstream microorganisms and toxins [33]. To confirm the penetration of H37Rv EVs through the BBB and assess their BBB-penetrating ability in vivo, we intravenously injected DiI-labeled H37Rv EVs into nude mice and captured near-infrared fluorescence images (Figure 7a,c). H37Rv EVs were detected in mouse brain tissue within 1 h of tail-vein injection, reaching peak fluorescence intensity at 12 h after administration, followed by a gradual decrease (Figure 7a,b). This suggests that H37Rv EVs can reach the brain within 1 h and accumulate to a maximum extent at 12 h, followed by gradual metabolic clearance. Additionally, ex vivo imaging of isolated brains clearly showed the distribution of DiI-labeled H37Rv EVs within the brain, although the fluorescence was slightly weaker than that observed in vivo (Figure 7c). Homogenized organ tissues showed that at 12 h, fluorescence intensity was 3.13 times higher in the liver and 1.56 times higher in the brain compared to the control. These findings further confirmed the entry of H37Rv EVs into the brain and liver, as well as their ability to cross the BBB (Figure 7d).

## 4. Discussion

The secretion of EVs is a conserved phenomenon in the mycobacterium genus, in both pathogenic and other mycobacterial strains (i.e., non-pathogenic and fast-growing strains) [34,35]. However, the variability in isolation methods can significantly affect the yield and purity of EVs, which is a critical consideration for downstream research. Mtb, with its thick cell wall, secretes EVs that may have unique characteristics and mechanisms [36]. The first step in researching *Mtb* EV functions involves isolating EVs that adhere to the 2023 Minimal Information for Studies of Extracellular Vesicles (MISEV) guidelines [37]. The composition and function of EVs vary across organisms and within the same organism depending on culture or life stage, and results from different EVs isolation methods may differ even for the same growth phases [6,38,39]. A reported method that combines ultrafiltration with size-exclusion chromatography has been found to yield up to 58 times more EVs and over double the amount of PD-L1-expressing EVs compared to ultracentrifugation, potentially enhancing immunomodulatory effects and furthering our comprehension of EVs [40]. The establishment of a mature, stable, convenient, and rapid method for isolating *Mtb* EVs is a prerequisite not only for explaining the conflicting findings but also for applying *Mtb* EVs in the fields of medical diagnosis, prediction, and pharmaceuticals; it is also important for a deeper understanding of the pathogenic mechanism of *Mtb*. The study reported in this paper is the first to comparatively assess the purity, particle size, and protein content of H37Rv EVs isolated using the DC, EI, and EXODUS methods, and then conduct an initial exploration of the BBB penetration ability and immune functions of these EVs.

The international standards for EVs isolation remain non-unified. Ultracentrifugation, the prevalent EVs isolation method, is costly, time-consuming, and yields low recovery with reduced specificity, often co-purifying bacterial components [41]. Ultrafiltration and SEC effectively separate EVs but can isolate similarly sized particles, compromising purity [42]. Immune capture isolation provides high specificity, purity, and convenience while maintaining EVs structure, but its reliance on positively marked EVs diminishes extraction efficiency, and unless antibodies can be readily detached post-precipitation, it may compromise exosome integrity [43]. Thus, weighing the pros and cons of these methods is vital for downstream research, as they affect yield, purity, and integrity.

In light of these challenges, our study evaluated three EVs isolation methods. All three EVs isolation methods used in this study effectively isolated H37Rv EVs, which exhibited a spherical shape and were of normal size. The lowest yield of H37Rv EVs was obtained using the DC method (Table 1, Figure 3a), indicating a loss of EVs due to the multiple washing steps. The method that yielded the highest amount of EVs (Table 1, Figure 3a) was the EI method, which primarily relies on the interaction between EVs and the precipitant [44]. However, the quantities yielded using this method may potentially contain additional small molecular aggregate proteins, leading to an inaccurate determination of EVs protein and particle concentrations [45,46]. Therefore, the EI method for EVs isolation has several disadvantages, including long time requirements, high isolation costs, and low purity (Figure 1 and Figure 2). A recently emerged EVs isolation technique, EXODUS, utilizes a negative pressure oscillation system in conjunction with a double-coupled harmonic oscillation system that operates on a nanofiltration chip. This process swiftly eliminates impurities such as free nucleic acids and proteins from samples through nanoscale pores while capturing EVs, resulting in the purification and enrichment of the EVs [17]. In the present study, the H37Rv EVs isolated using EXODUS exhibited more *Mtb* components and demonstrated stronger cellular immunity than those isolated using the DC and EI methods (Table 1, Figure 3 and Figure 5), indicating greater utility for the downstream analysis and applications of H37Rv EVs.

EVs are currently receiving widespread attention because of their roles in bacterial-cell communication and various biological processes [47,48]. The immunogenic contents of the EVs function as pathogen-associated molecular patterns (PAMPs), which can be detected by pattern recognition receptors (PRRs) like Toll-like receptors (TLRs), thereby activating innate immune responses [48]. Mtb EVs are pivotal in cell signaling, carrying a diverse array of biomolecules such as microbe-associated molecular patterns, enzymes, and toxins that modulate the functions of recipient cells upon release [7]. In the downstream application study of H37Rv EVs, in line with previous studies [49], an enrichment of lipoproteins in EVs was observed, with consequent promotion of TLR2 pathway activation (Table S2, Figure 5). And it appears that TLR-2 is not the sole receptor capable of sensing *Mtb* EVs, as a detailed examination has revealed that TLR-8 also becomes activated by mycobacterial RNA released within phagosomes by *Mtb* EVs, ultimately resulting in increased *Mtb* elimination through xenophagy, as documented in [50]. These studies inspire us to investigate the progression of TB from the vantage point of EVs.

Given the immunogenic contents of H37Rv EVs, we further investigated their impact on the central nervous system (CNS). The inflammation response induced by H37Rv EVs in the mouse brain was also investigated. At 3- and 6-days post-injection, degeneration, intense staining, widened intercellular spaces, and indistinct nuclei were observed in the cerebral parenchyma, cerebellum, and hippocampus. Notably, these changes were less pronounced on day 10 compared to day 0, as illustrated in Figure 6. Inflammation of the brain caused by H37Rv EVs may be due to the ability of EVs to penetrate the BBB [51] and load various PAMPs across the BBB, thus triggering an inflammatory response in the brain [52,53]. Our study confirmed that H37Rv EVs cross the BBB to reach the mouse brain within 1 h, with a subsequent peak at 12 h, followed by a gradual decrease in concentration (Figure 7). In addition, we also found that DiI-labeled H37Rv EVs are distributed not only in the brain but also in other organs, such as the liver, and the amount of H37Rv EVs in the liver is significantly higher than that in the brain. This may be because the liver is the largest metabolic organ in animals.

While our study confirmed the ability of H37Rv EVs to cross the BBB and induce an inflammatory response, the specific manner in which H37Rv EVs impact CNS disorders after crossing the BBB and entering the brain remains unclear. This is an area that requires further investigation in future studies. Previous research has demonstrated that EVs can affect CNS disorders after crossing the BBB through various mechanisms. Upon entry into the brain, the EVs derived from *Helicobacter pylori* EVs can be internalized by astrocytes, leading to glial cell activation, neuronal dysfunction, heightened beta-amyloid pathology, and cognitive decline, suggesting a possible role for EVs in the pathogenesis of Alzheimer’s disease (AD) [54]. *Porphyromonas gingivalis* (*Pg*) EVs, armed with gingipains on their surface, are adept at compromising the integrity of the BBB and contributing to the pathogenesis of AD [55,56]. Additionally, EVs can also fuse with *Streptococcus pneumoniae*, group B *Streptococcus*, and neonatal meningitis *Escherichia coli* to hitchhike on transferrin receptor (TfR) transcytosis to cross the BBB and be linked to meningitis [57]. These mechanisms offer guidance for the pathogenesis of tuberculous meningitis (TBM) and inform potential therapeutic strategies.

## 5. Conclusions

This study characterized the distinctive features of H37Rv EVs isolated by DC, EI, and EXODUS, with EXODUS identified as a convenient and dependable approach for EVs isolation, particularly well suited for downstream applications. H37Rv EVs were found to activate TLR2 signaling and provoke inflammation in RAW264.7 cells and can breach the BBB to initiate brain inflammation. These findings shed new light on the pathogenesis of *Mtb*-induced CNS disorders and suggest that EVs may serve as potential targets for treatments to prevent BBB breach and brain inflammation in tuberculosis.

## Figures and Tables

**Table 1:** Statistical analysis of the differences in particle size of H37Rv EVs isolated via different isolation methods.

Categories	DC	EI	EXODUS
Original Concentration (Particles/mL)	6.0 × 10^10^	3.1 × 10^11^	8.2 × 10^10^
Dilution Factor	500	2500	2000
Concentration (Particles/mL)	1.2 × 10^8^	1.3 × 10^8^	4.1 × 10^7^
Diameter/nm	116.7	137.2	110.3
X_10_	92.5	97.6	77.3
Median (X_50_)	135.0	149.4	112.4
X_90_	196.4	223.9	181.9

## Data Availability

The data presented in this study are available in the Supplementary Materials.

## Supplementary Materials

The following supporting information can be downloaded at: https://www.mdpi.com/article/10.3390/microorganisms12112129/s1. Figure S1: Nanoparticle tracking analysis of H37Rv EVs isolated by different methods. Figure S2: The original gel scans of Coomassie brilliant blue staining and Western blotting. Table S1: The primer sequences used in this study. Table S2: Mass spectrometry of *Mtb* EVs isolated using EXODUS. Table S3: The protein content of H37Rv EVs. Table S4: Cell viability of RAW264.7 cells after co-incubation with three types of H37Rv EVs for 24 h at 5 μg. Table S5: Q-PCR of *TLR2*, *IL-6* and *TNF-α* in RAW264.7 cells infected by three types of H37Rv EVs for 24 h at 5 μg. Table S6: ELISA of IL-6 and TNF-α in RAW264.7 cells infected by three types of H37Rv EVs for 24 h at 5 μg. Table S7: Fluorescence acquisition values of brain. Table S8: Fluorescence acquisition values of homogenized brain and liver.
