# Supplementary material for: Improved Isolation Optimizes Downstream Application of Extracellular Vesicles Derived from Mycobacterium tuberculosis
Source: Microorganisms. 2024 Oct 24;12(11):2129. doi: 10.3390/microorganisms12112129 (PMC11596817; doi:10.3390/microorganisms12112129)
Supplement: Supplementary file 1 [file microorganisms-12-02129-s001.zip › Supplementary Materials.pdf]

a

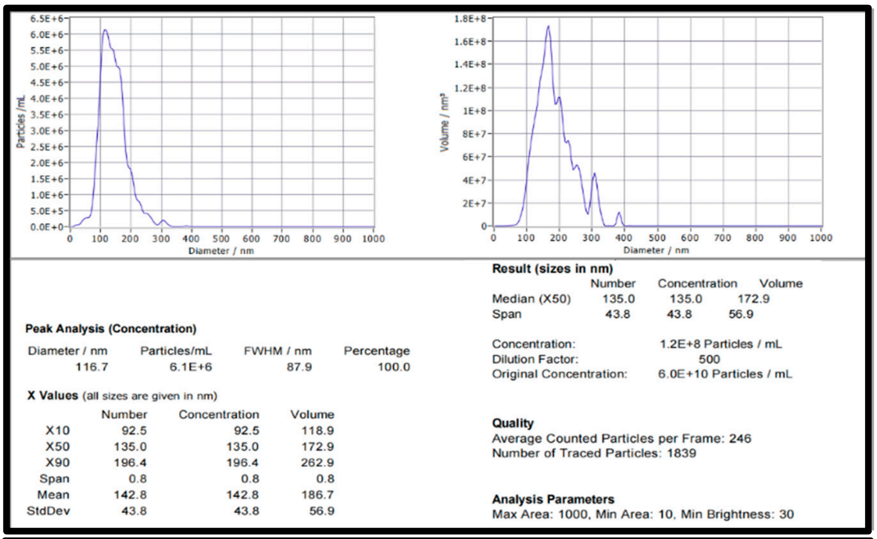

b

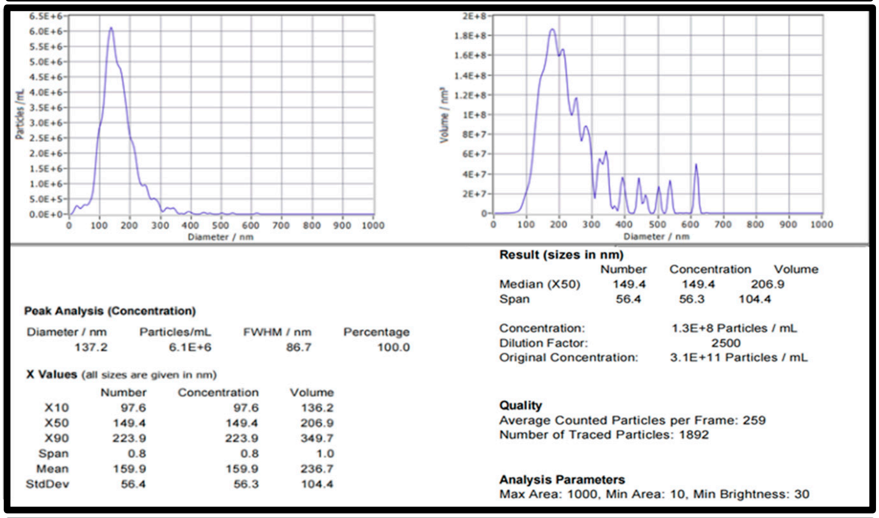

c

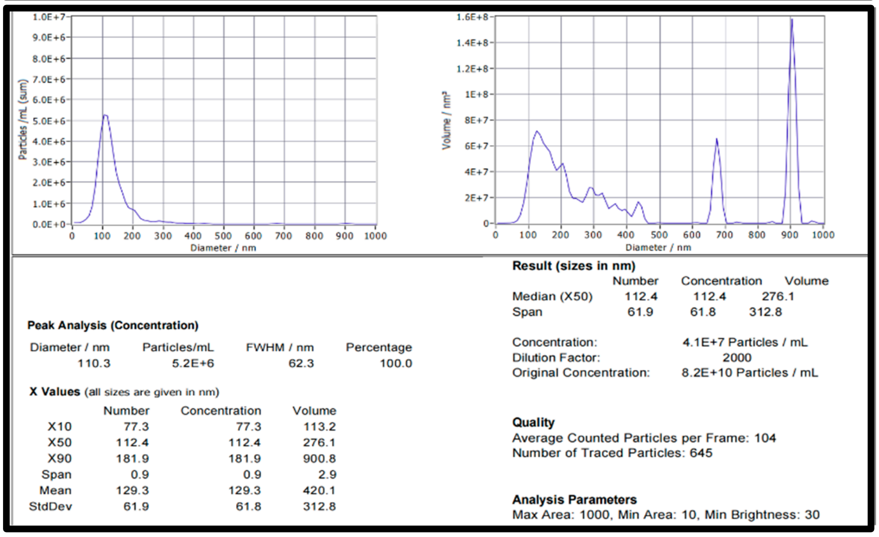

Figure S1: Nanoparticle Tracking Analysis report of H37Rv EVs isolated by different method. (a) DC; (b) EI; (c) EXODUS.

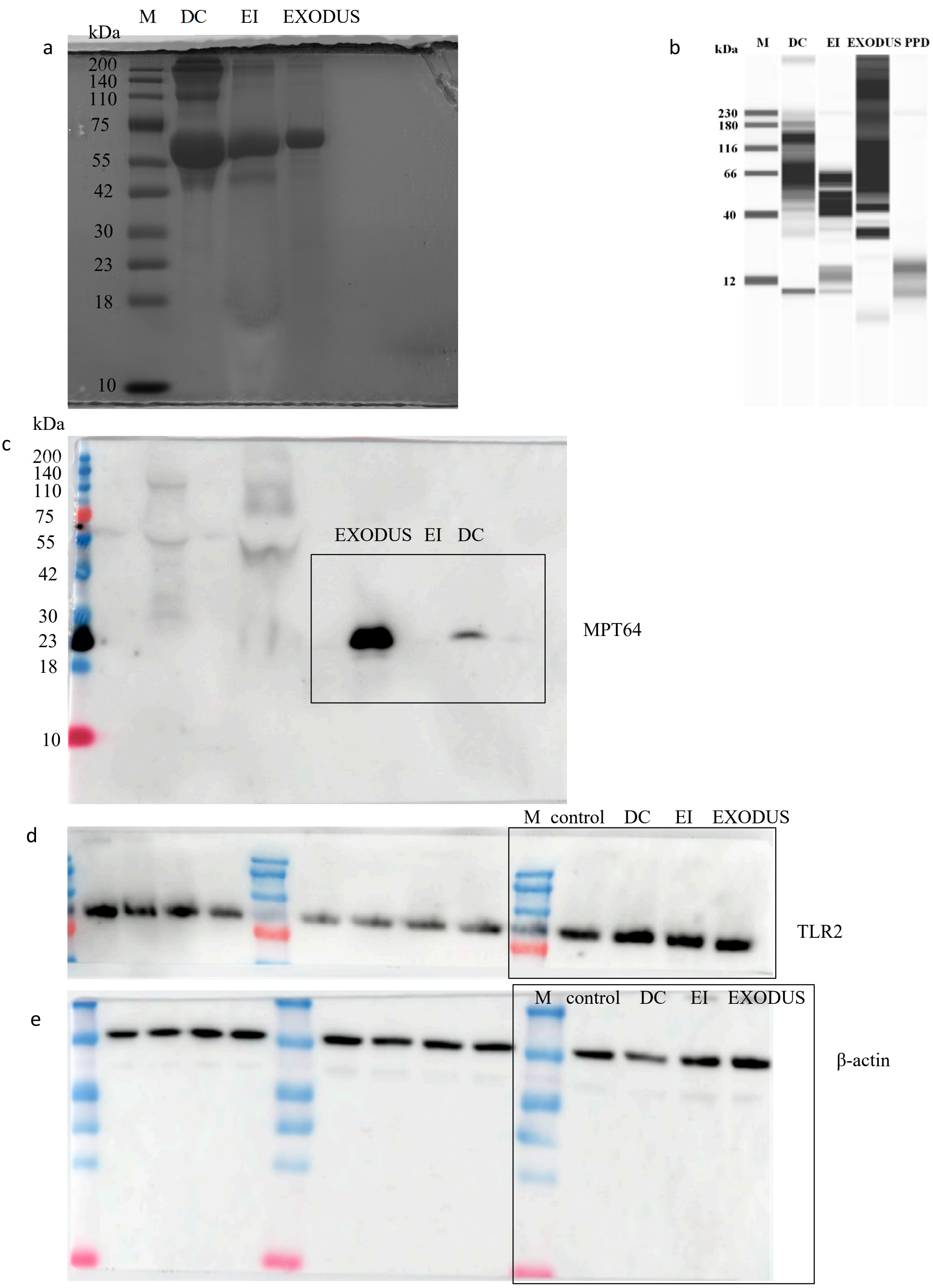
a
kDa
M DC EI EXODUS
200
140
110
75
55
42
30
23
18
10
b
kDa
M DC EI EXODUS PPD
230
180
116
66
40
12
c
kDa
200
140
110
75
55
42
30
23
18
10
EXODUS EI DC
MPT64
d
M control DC EI EXODUS
TLR2
e
M control DC EI EXODUS
β-actin

f

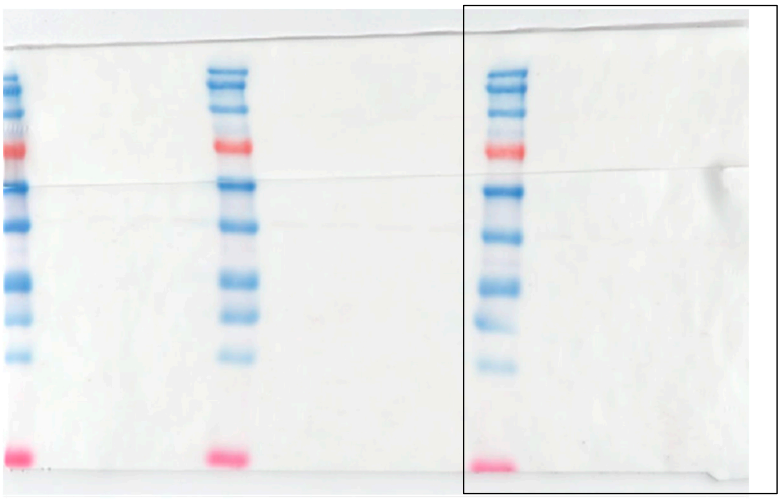

Figure S2: The original gel scans of coomassie brilliant blue staining and western blotting. (a) Coomassie brilliant blue image of three types of EVs; (b) Western blot image of PPD of three types of EVs; (c) Western blot image of MPT64 of three types of EVs; (d) Western blot image of TLR2 after three types of EVs (5 μg) infection of Raw264.7 for 4 h; (e) Western blot image of β-actin after three types of EVs (5 μg) infection of Raw264.7 for 4 h; (f) Complete western blot image of TLR2 and β-actin. M: protein marker; control: Raw264.7; DC: H37Rv EVs isolated by Differential Centrifugation; EI: H37Rv EVs isolated by Exosome Isolation kit; EXODUS: H37Rv EVs isolated by Exosome detection via the ultrafast-isolation system.

Table S1: The primer sequences used in this study

**Gene name**	**Gene Bank accession number**	**Primer**	**Sequences (5’⟶ 3’)**
**TLR2**	**NM_011905**	**Forward Primer**	CTCTTCAGCAAACGCTGTTCT
		**Reverse Primer**	GGCGTCTCCCTCTATTGTATTG
**IL-6**	**NM_031168**	**Forward Primer**	CTGCAAGAGACTTCCATCCAG
		**Reverse Primer**	AGTGGTATAGACAGGTCTGTTGG
**TNF-α**	**NM_013693**	**Forward Primer**	CCTGTAGCCCACGTCGTAG
		**Reverse Primer**	GGGAGTAGACAAGGTACAACCC

Table S3: The protein content of H37Rv EVs

	**Protein content (mg)**		
**DC**	1.425	1.3	1.5
**EI**	8.75	8.2	8
**EXODUS**	1.35	1.45	1.65

Table S4. Cell viability of Raw264.7 cells after co-incubation with three types of H37Rv EVs for 24 h at 5 μg.

OD_{450}		24h	
**RAW264.7**	1.19	1.12	0.90
**Differential Centrifugation**	0.59	0.57	0.46
**Exosome Isolation Kit**	0.62	0.61	0.66
**EXODUS**	0.69	0.57	0.59

Table S5. Q-PCR of TLR2, IL-6, TNF-α in RAW264.7 cell infected by three types of H37Rv EVs for 4 h at 5 μg.

Types of Mtb EVs	Compare categories		TLR2			IL-6			TNF-α	
**Differential Centrifugation**	Ct	24.78	24.50	24.80	25.84	25.69	25.88	21.76	20.99	21.10
	$2^{-\Delta\Delta Ct}$	1.35	1.24	1.33	424.48	470.75	414.68	2.40	4.11	3.81
	P value		*P*<0.01			*P*<0.0001			*P*<0.05	
	Up or Down		Up			Up			Up	
**Exosome Isolation Kit**	Ct	23.99	23.57	24.30	25.97	26.34	26.16	20.94	20.97	20.99
	$2^{-\Delta\Delta Ct}$	1.77	2.37	1.43	295.22	228.98	259.19	3.22	3.16	3.11
	P value		*P*<0.05			*P*<0.001			*P*<0.0001	
	Up or Down		Up			Up			Up	
**EXODUS**	Ct	24.98	24.47	24.29	25.76	26.03	26.34	20.42	20.49	19.91
	$2^{-\Delta\Delta Ct}$	1.46	2.08	2.35	560.78	464.39	374.55	7.56	7.19	10.75
	P value		*P*<0.05			*P*≤0.001			*P*<0.01	
	Up or Down		Up			Up			Up	

Table S6. ELISA of IL-6, TNF-α in RAW264.7 cell infected by three types of H37Rv EVs for 4 h at 5 μg.

Expression of IL-6/TNF-α		IL-6			TNF-α	
**Raw264.7**	2.32	2.66	4.09	264.56	260.89	265.79
**Differential Centrifugation**	673.78	682.04	617.94	3446.43	3156.64	3231.34
**Exosome Isolation Kit**	427.90	433.26	459.74	3765.06	4446.41	2869.49
**EXODUS**	695.76	700.55	668.31	4766.22	5040.23	4892.98

Table S7: Avg Radiant Efficiency values of brain

Radiant Efficiency values		brain (10^7)	
**1 h**	1.76	1.56	1.32
**4 h**	2.08	1.39	1.83
**8 h**	2.06	1.50	1.92
**12 h**	1.85	1.86	2.04
**24 h**	1.58	1.39	1.68

Table S8: Fluorescence acquisition values of homogenised brain and liver

Fluorescence acquisition values	brain			liver		
**blank**	453	494	643	1062	3398	2048
**8 h**	528	598	674	4161	5716	3811
**12 h**	1065	678	729	7896	7513	4946
**24 h**	568	571	649	2510	4125	2518
